# Selection for energy efficiency drives strand-biased gene distribution in prokaryotes

**DOI:** 10.1038/s41598-017-11159-3

**Published:** 2017-09-05

**Authors:** Na Gao, Guanting Lu, Martin J. Lercher, Wei-Hua Chen

**Affiliations:** 1 Key Laboratory of Molecular Biophysics of the Ministry of Education, Hubei Key Laboratory of Bioinformatics and Molecular-imaging, Department of Bioinformatics and Systems Biology, College of Life Science and Technology, Huazhong University of Science and Technology (HUST), 430074 Wuhan, Hubei China; 2 Department of Blood Transfusion, Tangdu Hospital, the Fourth Military Medical University, No 1, Xinsi Road, Chanba District, 710000 Xi’an, China; 3 Institute for Computer Science and Cluster of Excellence on Plant Sciences CEPLAS, Heinrich Heine University, 40225 Düsseldorf, Germany

## Abstract

Lagging-strand genes accumulate more deleterious mutations. Genes are thus preferably located on the leading strand, an observation known as strand-biased gene distribution (SGD). Despite of this mechanistic understanding, a satisfactory quantitative model is still lacking. Replication-transcription-collisions induce stalling of the replication machinery, expose DNA to various attacks, and are followed by error-prone repairs. We found that mutational biases in non-transcribed regions can explain ~71% of the variations in SGDs in 1,552 genomes, supporting the mutagenesis origin of SGD. Mutational biases introduce energetically cheaper nucleotides on the lagging strand, and result in more expensive protein products; consistently, the cost difference between the two strands explains ~50% of the variance in SGDs. Protein costs decrease with increasing gene expression. At similar expression levels, protein products of leading-strand genes are generally cheaper than lagging-strand genes; however, highly-expressed lagging genes are still cheaper than lowly-expressed leading genes. Selection for energy efficiency thus drives some genes to the leading strand, especially those highly expressed and essential, but certainly not all genes. Stronger mutational biases are often associated with low-GC genomes; as low-GC genes encode expensive proteins, low-GC genomes thus tend to have stronger SGDs to alleviate the stronger pressure on efficient energy usage.

## Introduction

In most prokaryotic genomes, protein-coding genes are preferably located on the leading strand^1^, on which the replication is continuous^2^. For example, in contrast to randomly expected 50% if there were no strand preferences, over 90% of the 1,552 bacterial and archaeal genomes we surveyed in this study show preferred location of their coding genes on the leading strand (see also^3^). This phenomenon, which is known as biased-strand gene distribution (SGD), has been intensively investigated in the past decades and many hypotheses have been proposed^4–15^.

It has long been suspected that SGDs are caused by collisions between the replication and transcription machineries^1, 4, 9, 11, 14–17^. The latter two share the same DNA template but move with different speed^6^; in addition, they move in different directions on the lagging strand of the genome. Thus, collision can happen either co-directionally (on leading strand) or head-on (on lagging strand)^16^. Collisions can cause replication stalling, abortive transcription, and expose single-stranded DNAs to chemical modifications and other damages^18^. Collisions are thus deleterious. Recent experimental results suggest that genes on the lagging strand accumulate more mutations than those on the leading strand^19^, due to head-on collisions or the discontinuous nature of the DNA synthesis of the lagging strand, or both. This indicates that head-on collisions are more deleterious than co-directional collisions. The elevated deleterious effects on the lagging strand are believed to cause a higher burden on fitness for highly expressed genes and functionally important genes (*e.g*., essential genes), consistent with the observations that these two types of genes are underrepresented on the lagging strand^9, 12^.

**Figure 1:**
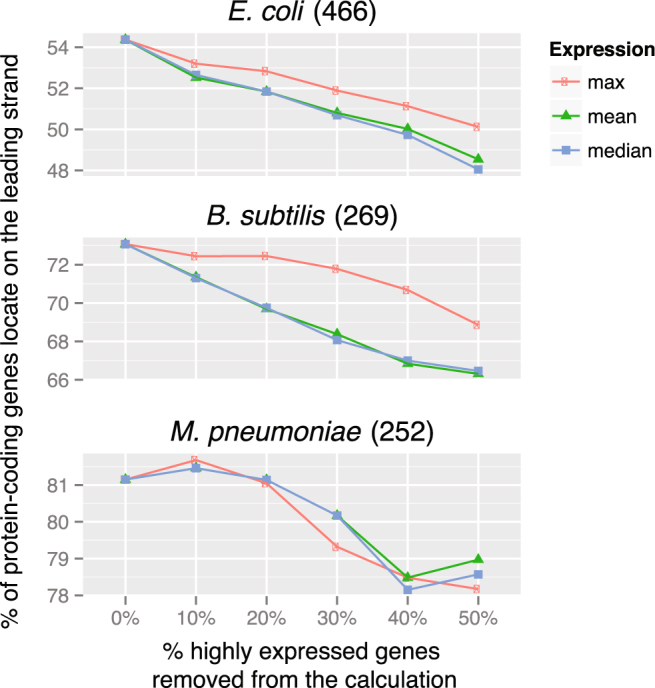
Removing highly expressed genes does not eliminate strand-biased gene distribution in selected species. Gene expression data were downloaded from NCBI GEO database^36^ for the three model bacteria, *Escherichia coli* ^37^, *Bacillus subtilis* ^38^ and *Mycoplasma pneumoniae* ^34^; the number of datasets for each species is indicated in the parenthesis of the panel title. For each gene in a genome, we calculated the max, mean and median expression values across the expression datasets we collected, and then ranked all genes in a genome accordingly.

**Figure 2:**
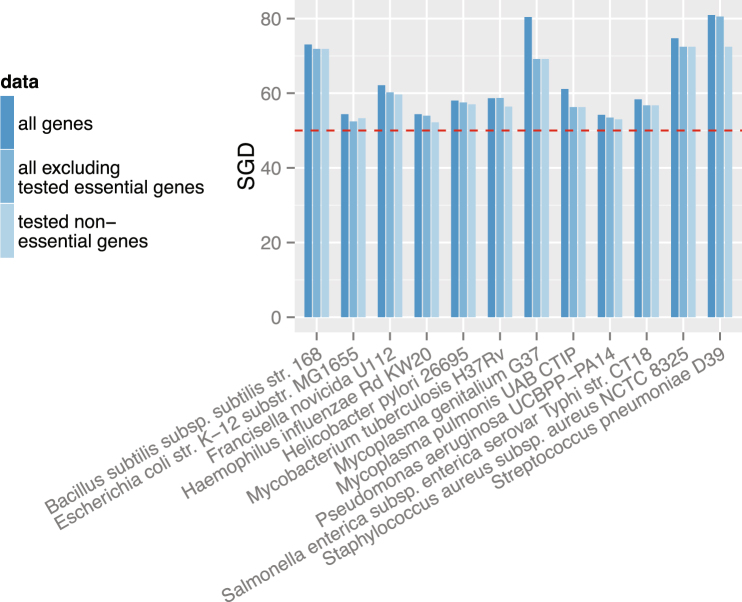
Removing essential genes does not eliminate strand-biased gene distribution in selected species. Tested essential and nonessential genes were obtained from OGEE - an online gene essentiality database^25^. “all genes” (dark blue bar): when all genes were used to calculate the SGD; “all excluding tested essential genes” (blue bar): when genes that were tested as nonessential genes and those were not tested in gene essentiality experiments were used; “tested non-essential genes” (light blue bar): when only genes that were tested as nonessential were used.

Despite the mechanistic insights, a quantitative model that explains the variation of SGDs in different species is still lacking. For example, the expression-driven^9^ and essentiality-driven^12^ hypotheses are not quantitative; more importantly, after highly expressed and essential genes were removed, SGDs were decreased but not completely removed (see Figs 1 and 2). In addition, it is difficult to quantify their contributions to SGD: it is unclear why SGDs are different in different genomes, and how much of the variations can be explained by essential or highly expressed genes. Recently, Mao *et al*.^3^ proposed a very sophisticated model; using data on the enrichment and depletion of genes in 25 Gene Ontology (GO) categories on the leading strand, they were able to explain ~74% of the variance of SGDs across 725 prokaryotic genomes; the authors argue that genes of certain functions prefer different strands and consequently drive SGD. Although it represents arguably one of the best quantitative models so far, ref. 3 blurs the cause and consequences of this issue. For example, one may argue that it is the head-on collisions between replication and transcription machineries that drive the highly-expressed and essential genes to the leading strand, and consequently cause the biased functional categories in the genes on the leading strand, rather than the other way round.

Here, we propose a mutagenesis/energy efficiency model for SGDs and test it on 1,552 prokaryotic genomes. In previous work, we showed that strand-specific mutational biases, observed as nucleotide compositional biases in inter-operonic regions, can be recapitulated using coding sequences from leading and lagging strands^20^. These results suggested that mutational biases in coding regions are of similar nature to that in non-transcribed regions but are inflated, likely due to the longer exposure time of single-stranded DNA during transcription^20^, which causes increased DNA damage and error-prone repair. Mutational biases introduce the energetically cheaper nucleotides *T* and *C* over their complementary nucleotides *A* and *G*, respectively, as well as *C* over *G* on the lagging strand. Due to a trade-off between nucleotide and amino acid costs inherent in the codon translation table, the bias towards cheaper nucleotides results in more expensive protein products for genes on the lagging strand, driving genes to the leading strand.

Our model – which we develop in quantitative form below – makes the following predictions. First, strand-specific mutational biases observed in interoperonic regions should be able to predict the extent of SGD in a given genome: stronger mutational biases should lead to stronger SGD. Second, previous studies have shown that costs per protein decrease with increasing gene expression^20–24^; therefore, highly expressed genes on the lagging strand should still be cheaper than lowly expressed genes on the leading strand. We thus expect selection for energy efficiency to drive some genes to the leading strand, especially those highly expressed and essential, but not all genes.

## Results and Discussion

### Removing highly expressed or essential genes does not eliminate SGD

Avoidance of head-on collisions between replication and transcription machineries could drive (some) highly-expressed and/or essential genes to the leading strand. However, we hypothesized that other factors such as mutagenesis could also contribute significantly to SGDs. We thus removed highly expressed or essential genes from selected species and recalculated SGDs. As expected, SGDs remain in most species, especially in genomes with strong SGDs to begin with, suggesting that highly expressed or essential genes could only explain a small part of SGD (Figs 1 and 2).

**Figure 3:**
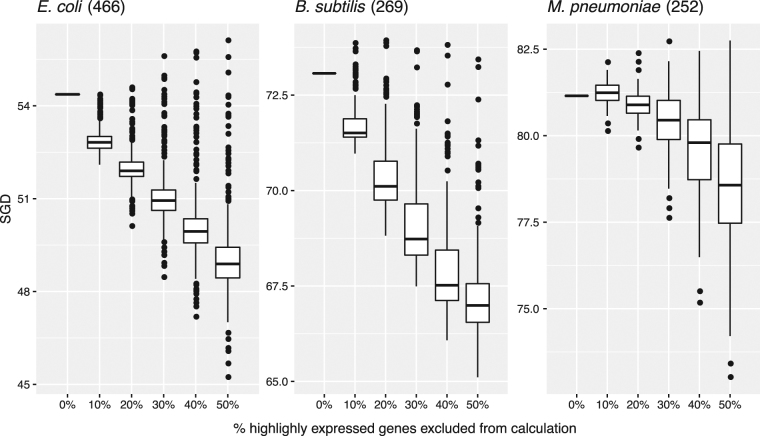
SGDs decrease but remain after removing highly expressed genes in selected species. The same data from Fig. 1 were also used here. For each expression dataset, we ranked genes according to their expression abundances, removed the highly-expressed ones and recalculated the SGD. We summarized the results as boxplots.

Gene expression abundances vary between different experimental conditions. We thus also tested whether the same trend could be observed in individual gene expression experiments. From each of the expression datasets we collected for the selected organisms, we ranked genes according to their expression abundance, removed the highly-expressed ones and recalculated the SGD. Figure 3 summarized the results as boxplots; as expected, we observed the same trend that SGDs decrease but remain after removing highly expressed genes.

Gene essentiality statuses can also be environment-/experiment-dependent. We thus further tested our hypothesis in species whose essential genes had been tested under different experimental conditions. As shown in Supplementary Figure 1, in all four bacteria (namely *Salmonella enterica subsp. enterica serovar Typhimurium str. SL1344*, *Pseudomonas aeruginosa UCBPP-PA14*, *Escherichia coli K12* and *Mycobacterium tuberculosis H37Rv*) for which multiple essentiality datasets are available in OGEE v2^25^, removing essential genes did not eliminate SGD.

**Figure 4:**
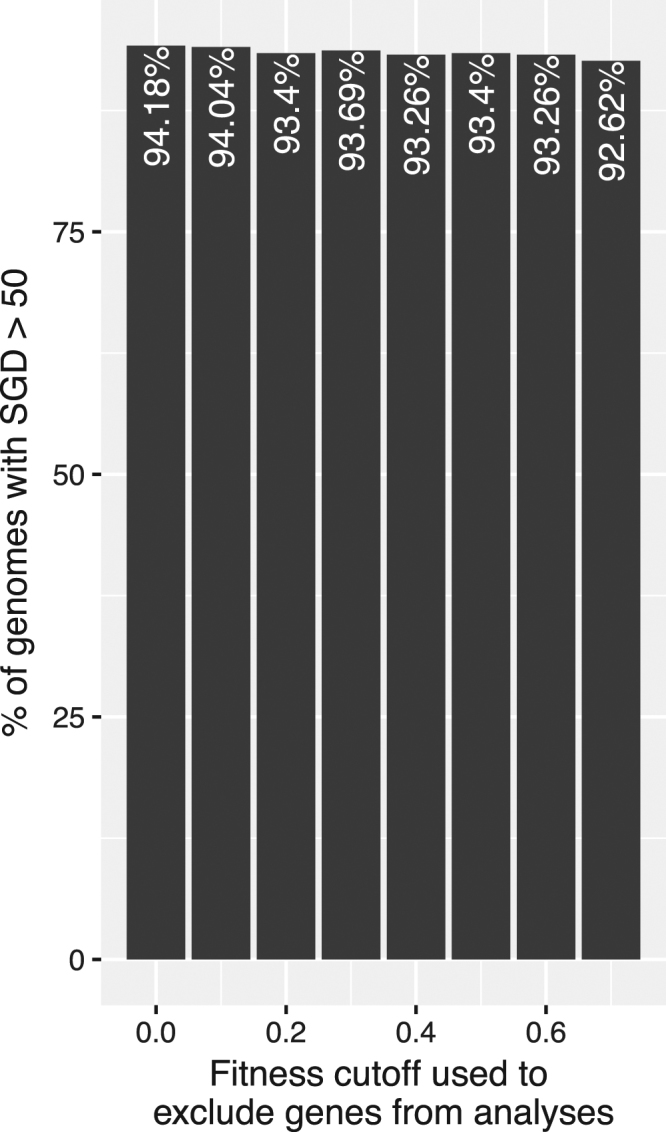
Excluding essential genes does not eliminate SGDs using quantitative measurements of gene essentiality (Fitness scores) obtained from IFIM, a database of Integrated Fitness Information for Microbial genes^27^. Genes with lower fitness scores more likely to be essential.

Gene essentiality can also be measured quantitatively (*e.g*., as Fitness scores) instead of qualitatively; it has been previously shown that quantitatively measured gene essentiality contributes significantly to SGD in bacterial species^26^. To further test the robustness of hypotheses on this type of data, we obtained predicted “fitness scores” for 2,074 species from IFIM, a database of Integrated Fitness Information for Microbial genes^27^. Fitness scores in IFIM were predicted using Geptop^28^ based on orthology and phylogeny; the scores range from 0 to 1, with lower scores representing greater fitness decreases and thus higher likelihood of being essential. A cutoff of 0.65 was recommended to classify genes into essential (those with fitness scores <= 0.65) and non-essential^27, 28^. In total, 1,410 genomes overlapped with the 1,552 genomes used in this study. As shown in Fig. 4, when all genes were included, ~94.18% of the 1,410 genomes had SGDs larger than 50; excluding genes with lower fitness scores could reduce this percentage, but only to a very limited extend. For example, after excluding genes with fitness scores less than 0.7 from all genomes and re-calculating SGD, 92.62% of the genomes still had SGDs larger than 50.

Together, these results further confirmed that highly expressed or essential genes could only explain part of SGD in prokaryotes.

### Replication skews can explain ~71% of the variance in SGDs in 1,552 prokaryotic genomes

Our previous results showed that mutational biases, i.e. strand-specific usage of *A* versus *T*, and of *G* versus *C* (also known as AT and GC skews respectively; see Methods) observed in interoperonic regions can be recapitulated using coding sequences from leading and lagging strands, with a certain inflation^20^. For example, mutational skews estimated by contrasting genes on the leading strand and on the lagging strand correlate significantly with the interoperonic skews, with correlation coefficients of 0.78 and 0.90 for AT and GC skews, respectively. Interoperonic regions are either non-transcribed or only casually transcribed^29^, and their skews are thus predominantly due to mutational biases and not to natural selection (see also ref. 20). These results indicate that mutational biases in coding regions are of a similar nature as those in non-transcribed regions; the inflation was likely due to the prolonged exposure time of single-stranded DNA during transcription and replication-transcription-collisions^20^, followed by increased DNA damage and error-prone repair.

It has long been suspected that there is a connection between SGDs and the mutational biases^4, 30^. For example, Hu and colleagues found that the nucleotide skews at fourfold-synonymous (4 s) sites of the coding regions and in intergenic regions correlate significantly with SGD (Pearson’s correlation coefficients *R* > 0.7 in both cases)^4^. One problem with this calculation is the inclusion of transcribed regions. It is known that the overall nucleotide skews of the transcribed regions consists of at least two parts, one part is attributed to replication (i.e. mutational biases), while the other is attributed to transcription^20^. The replication skews in transcribed regions are proportional to that in interoperonic regions but slightly inflated, with the inflation rate being proportional to expression abundance^20^. Genes on the leading strand are often more abundantly expressed; the stronger the SGDs, the stronger the differences in expression abundances between strands, and the stronger the differences in nucleotide skews. Therefore, the inclusion of coding/transcribed regions in Hu’s calculation will inflate the correlation by partially correlating SGD with its consequences (Methods).

**Figure 5:**
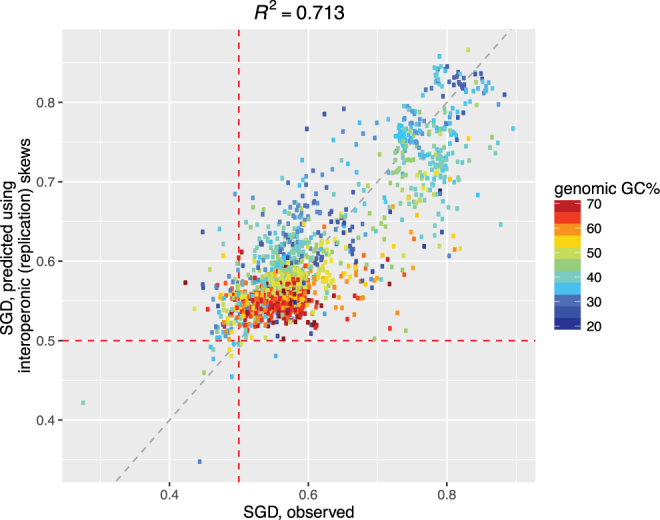
Predicted SGDs (y-axis) in 1,552 bacterial genomes using interoperonic skews and their correlation with the observed SGDs (x-axis). Each dot represents a genome, color-coded by genomic GC-content.

By using a simple nonlinear regression model (Multivariate adaptive regression splines, MARS; Methods) on the interdependence of SGD and mutational bias (Fig. 5), we estimated that ~71% of the variation in SGDs in 1,552 prokaryotic genomes can be explained by the nucleotide skews from interoperonic regions that are presumably only subjected to replication (we hence refer them as replication skews; see also the discussions below) (Fig. 5). Our model has similar predictive power as the model proposed by Mao and colleagues (Pearson’s *R* ^2^ 71% versus 74%) but uses much fewer variables as input (2 versus 28)^3^; more importantly, SGD and replication skews in our model were derived from non-overlapping datasets. Our model thus clearly indicates that SGD and replication skews may have a common origin*, i.e*., the factors that drive replication skews also drive SGD; the stronger the replication skews, the stronger the SGD (Fig. 5). Consistent with our expectations, the inclusion of coding / transcribed regions into the calculation indeed inflated the correlation: we estimate that over ~78% of variations in SGDs could be explained by the overall nucleotide skews (Supplementary Figure 3).

### Mutational biases cause the use of slightly more expensive amino-acids in genes on the lagging strand

The synthesis of the four nucleotides *A, C, G, T* requires different amounts of energy: *de-novo* production costs are *A* > *T*, *G* > *C*, and *G* + *C* > *A* + *T* ^20^. Replication skews are strand-specific; the leading strand is biased towards the more expensive nucleotide *G* over *C* in almost all prokaryotic genomes (93.9%), while on the lagging strand the opposite is found. Although only a small proportion of prokaryotes (36.1%) preferentially use the more expensive nucleotide *A* over *T*, a majority (87.6%) of the collected genomes prefer the use of the more expensive purines (*G* and *A*) over pyrimidines (*T* and *C*) on the lagging strand in interoperonic regions (Supplementary Table 1).

**Figure 6:**
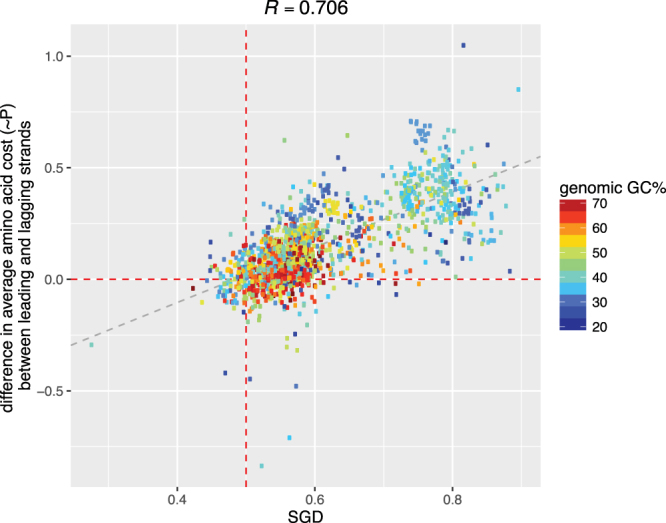
correlation between strand-biased gene distribution (SGD; x-axis) and the difference of average costs per amino acid of gene products encoded by genes on the lagging and leading strand.

Replication skews also exist in coding regions, where they are inflated as a function of expression abundance^20^. Due to an intrinsic tradeoff in the codon table, more expensive nucleotides code for cheaper amino acids and *vice versa* ^20^; we thus expect that the replication skews would cause slightly cheaper protein products on the leading strand. This is indeed the case: we found that 91% of the genomes with positive purine skews (that is, purines are preferred over pyrimidines) encode cheaper protein products on their leading strand; interestingly, 62.5% of genomes with negative skews (that is, pyrimidines are preferred over purines) also encode cheaper protein products on their leading strand, indicating that additional factors such as GC-content also contribute to these observations. In addition, we found that the protein cost differences between lagging and leading strands (*i.e*., average cost per amino acid of the lagging strand minus that of the leading strand) correlate significantly with replication skews (Pearson’s *R* = 0.56, 0.47 and 0.61 for AT, GC, and the overall Purine-skews, respectively; see Methods) as well as with SGD (*R* = 0.701, Fig. 6).

Mutations are also known to be biased towards *AT* in bacteria^31^. Recent experimental results suggested that due to head-on collisions, lagging-strand genes tend to accumulate more mutations than leading-strand genes^19^ and thus have lower GC-contents and code for more expensive proteins than leading-strand genes. A nonlinear regression analysis using MARS revealed that both the replication skews and the overall differences in GC-content between leading and lagging strand genes contribute significantly to the amino acid differences, with the replication skews as the most important factor, followed by GC-differences. Similarly, a linear regression model implemented in the R package ‘relaimpo’ reported that the replication skews contributed twice as much as the GC-differences (Methods). These results suggest that the protein cost difference between the two strands can be mostly attributed to replication skews.

### Selection for energy efficiency drives some, but not all highly expressed genes to the leading strand

**Figure 7:**
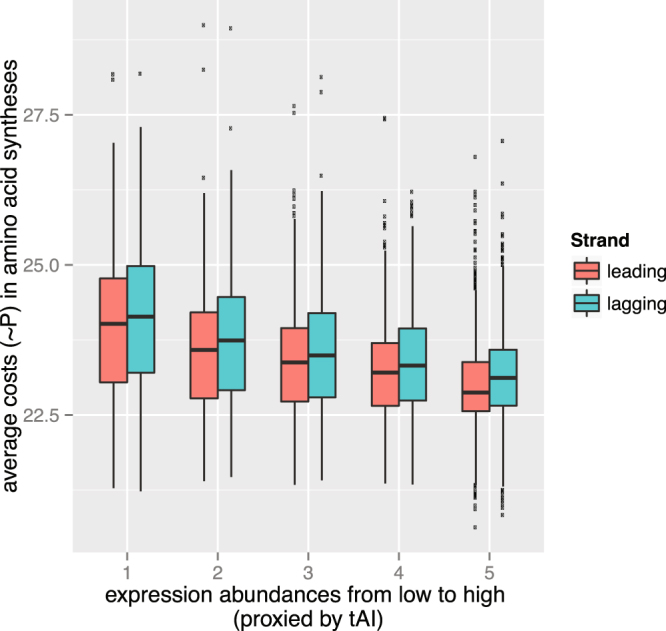
average costs in amino acid synthesis as a function of leading/lagging strand and expression abundance. Genes in each genome were ranked according to their expression abundance (proxied by tAI, tRNA adaption index) from low to high, divided into five equal-sized bins (so that each bin contains roughly the same number of genes) and then divided into two sub-groups according to their strand (leading versus lagging).

As shown in Fig. 7, when expression abundances (proxied by tAI, tRNA adaptation index^32, 33^) are similar, protein products are always slightly more expensive on the lagging strand; however, as the per protein costs decrease with increasing expression abundance due to increasing skews^20^ and GC-contents (see also Supplementary Figure 4), the protein products of lowly expressed leading strand genes could be more expensive than those of highly expressed lagging strand genes. These results have two important implications. First, for the purpose of energy efficiency, there is a tendency for highly expressed genes, especially those that are also universally expressed, to move to the leading strand through the fixation of local chromosomal inversions. This would explain why genes such as those involved in transcription, translation, and replication are preferably located on the leading strand; this would also increase the ratio of essential genes on the leading strand because these genes are more likely to be essential. Second, there is no need to move all genes to the leading strand. In fact, it might be beneficial to distribute genes onto different strands, *e.g*., to avoid possible “transcriptional leakage” if transcription termination fails accidentally. This is consistent with a previous observation that more “unbalanced genomes”, *i.e*., those with strong SGDs, tend to have longer intergenic regions^3^ in order to give more space or harbor necessary *cis*-regulatory elements and sequence signatures for the transcription machinery to terminate properly.

### Relationships between mutational bias, GC-content, and genome size

Interestingly, we found that the genomic GC-content correlates significantly with both AT and GC replication skews (*R* = −0.32 and −0.54 for AT and GC skews, respectively, *P* < 2.2 × 10^−16^; AT and GC skews are also significantly correlated with each other, consistent with recent studies^30^). Because *G* + *C* are more expensive than *A* + *T* and encode cheaper amino acids, high-GC genomes spend proportionally more energy on nucleotide production than low-GC genomes, while the latter spend relatively more energy on the production of amino acids; in other words, genomic GC-content is an indicator of relative energy investment into nucleotides and amino acids^20^. GC content also correlates with genome size^20, 34^. As amino acids are relatively more expensive than nucleotides (Supplementary Table 2, see also ref. 20), the selection for energy efficiency is stronger in low-GC genomes. The negative correlation between the replication skews and genomic GC indicates that stronger (more positive) replication skews are preferentially found in low-GC genomes and could result in cheaper encoded amino acids, thus partially alleviating the strong selection pressure due to low GC. These results suggest that replication skews are also influenced by selection for energy efficiency.

Intracellular pathogens and symbionts spend their entire life cycle inside the cells of other organisms that are often much larger in size; in other words, they live in extremely nutrient-rich environments and thus experience weaker selection on efficient resource usage^20^. Excluding 126 previously identified intracellular pathogens and symbionts (Table S2) from our analyses improved the correlation between genome-GC and replication skews (*R* = −0.35 and −0.57 for AT and GC skews respectively). These results further supported our conclusion that selection for energy efficiency constrain replication skews.

### Relationship between our model and existing theories

Our model is compatible with many existing hypotheses. For example, similar to the head-on collision model, our model predicts that highly-expressed and essential genes are to be over-represented on the leading strand, consistent with previous observations^9, 12^. However, although the head-on collision model is not quantitative, it also predicts that important non-coding genes such as tRNA and rRNA genes should be preferably located on the leading strand. In addition, the head-on collisions alone could drive genes to the leading strand, by either causing abortive transcription of genes that should be stably expressed at all times (*e.g*., ribosomal genes), or introducing more deleterious mutations into the regulatory regions of genes, or both. Our model does not explicitly cover these situations.

A recent study by Paul *et al*. proposed that some lagging-strand genes take advantage of the increased mutagenesis resulting from the head-on collisions and are thus adaptively encoded on the lagging strand^17^. This model is the opposite to our model, and has been recently rebutted by Chen and Zhang^35^. Chen and Zhang reanalyzed the data in ref. 17 and found no evidence for adaptive evolution of the lagging-strand genes; instead, they argue that SGD can be explained by a mutation-selection balance model, where deleterious chromosomal inversions move genes from the leading to the lagging strand and purifying selection purges such mutants^35^, a view compatible with our model.

In this study, we proposed an energy efficiency theory for strand-biased gene distributions (SGD) and tested it on prokaryotic genomes. We showed that due to elevated mutational biases on the lagging strand, proteins encoded by lagging-strand genes are slightly more expensive than those encoded by leading-strand genes. Consequently, genes, especially those that are highly expressed, are preferentially located on the leading strand. Highly expressed genes code for cheaper products, even when they are located on the lagging strand; thus not all highly expressed genes, and certainly not all genes would be moved to the leading strand. Our model is compatible with many existing hypotheses and can explain more than two-third (~71%) of the variance in SGDs.

## Methods

Gene expression data were downloaded from NCBI GEO database^36^ for the three model bacteria *Escherichia coli* ^37^, *Bacillus subtilis* ^38^ and *Mycoplasma pneumoniae* ^34^. Gene essentiality data for selected model organisms were downloaded from OGEE – an online gene essentiality database^25^.

Genome sequences and annotation for all completely sequenced prokaryotes were downloaded from NCBI Genbank^39^. Genomic coordinates for replication starts were downloaded from DoriC^40^; replication ends were obtained by adding ½ genome lengths to the starts. This working definition of replication termination was inferred from the work of Hendrickson and Lawrence^41^, in which the authors found that replication in *E. coli* is more likely to terminate near the ½ genome length to the oriC site, instead of the multiple *Ter* sites in the genome (Fig. 1 of ref. 41). 1,552 genomes covered by all three databases were used in this study (Table S1). The division of a genome into leading and lagging strands is shown in Supplementary Figure 2. Coding genes located on the first half of the plus strand (blue solid line) and on the second half of the complementary strand (purple solid line) were assigned to the leading strand, as their transcription proceeds in the same direction as the replication fork; the remaining genes were assigned to the lagging strand.

Operon predictions were downloaded from DOOR^42^. Because the predictions only cover coding regions, we added other annotated regions including tRNAs and rRNAs from the GFF (General Feature Format) annotations downloaded from NCBI, so that we could extract interoperonic regions, which are presumably non-transcribed. To extract regions that are presumably only subject to replication, interoperonic sequences longer than 100 basepairs were retained after removing 60 bp from the regions adjacent to the 5′-end of genes/operons. If an interoperonic region was located in the second half of the genome (blue dashed line in Supplementary Figure 2), its sequence was reverse-complemented. Replication skews are denoted as *γ*_*AT*_ (for AT skew) and *γ*_*GC*_ (for GC skew) and were calculated using extracted interoperonic regions using the equations below:

1 $${\gamma }_{AT}=\frac{A-T}{A+T}$$

and

2 $${\gamma }_{GC}=\frac{G-C}{G+C}$$

where *A*, *T*, *G*, *C* are the numbers of the corresponding bases. The overall purine skews were also calculated similarly using the equation below:

3 $${\gamma }_{purine}=\frac{A-T+G-C}{A+T+G+C}$$

The costs of *de novo* amino acid synthesis were obtained from^21^ (Table S2). The costs of *de novo* nucleotide synthesis were obtained from^20^ and are 21.12, 13.42, 20.37, 15.77 ATPs for *A*, *T/U*, *G*, *C* respectively; please note these numbers were calculated for *E. coli* and might be different for other organisms.

tAI (tRNA adaptation index)^32, 33^ was used as a proxy for gene expression level. For each protein-coding gene in a given genome, tAI is defined as the average of tRNA availability values over all its codons. The availability of tRNAs for a codon considers not only the copy number of perfectly matched anticodons in the corresponding genome, but also that of imperfectly matched anticodons; the contribution of the imperfectly matched anticodons will be weighted accordingly. For more details on the definition of tAI see refs 32, 33. For each of the selected 1,552 genomes, we obtained a list of tRNA genes using the tRNAscan-SE^43^ program on the genome sequences. The tRNA genes were sorted into 61 groups according to their anticodons. We then used the R scripts for tAI calculation written by the authors of refs 32, 33 (obtained from http://people.cryst.bbk.ac.uk/~fdosr01/tAI/, without modifications) to calculate tAI scores for all protein-coding genes in this genome. Higher tAI scores indicate higher expression levels.

Within each genome, coding genes were ranked according to their tAI scores from low to high and then divided into five equal-sized bins (quantiles), denoted 1 to 5; 1 contains the genes with the lowest, and 5 contains the genes with the highest tAI scores. Genes in each bin were then further divided into two groups according to the strands (leading versus lagging) they are located on.

Fitness scores (i.e. quantitative measurements of gene essentiality) for 2,074 prokaryotic genomes were downloaded from IFIM, a database of Integrated Fitness Information for Microbial genes^27^. Fitness scores in IFIM were predicted using Geptop^28^ based on orthology and phylogeny; the scores range from 0 to 1, with lower scores representing greater fitness decreases and thus the corresponding genes are highly likely to be essential. A cutoff of 0.65 was recommended to classify genes into essential (those with fitness scores <= 0.65) and non-essential^27, 28^. In total, 1,410 genomes overlapped with the 1,552 genomes used in this study.

All data was analyzed in R^44^. Non-linear regression analyses were carried out using the MARS (multivariate adaptive regression splines) function implemented in the ‘earth’ package of R (available at: https://cran.r-project.org/web/packages/earth/index.html); linear modeling was done with the ‘relaimpo’ package^45^. All plots were generated using the ggplot2^46^ package.

## Electronic supplementary material

Supplementary Info

Supplemental Table 1

Supplemental Table 2
