# Supplementary material for: Selection for energy efficiency drives strand-biased gene distribution in prokaryotes
Source: Sci Rep. 2017 Sep 5;7:10572. doi: 10.1038/s41598-017-11159-3 (PMC5585166; doi:10.1038/s41598-017-11159-3)
Supplement: Supplementary file 1 — Supplementary Info [file 41598_2017_11159_MOESM1_ESM.doc]

# Supplementary info for:

**Selection for energy efficiency drives strand-biased gene distribution in prokaryotes**

Na Gao ^1, 3 *^, Guanting Lu ^2, *^ , Martin J Lercher ^3^, Wei-Hua Chen ^1§^

# Supplementary Figures

**Supplementary Figure 1.** Excluding essential genes does not eliminate SGD; shown here are species whose essential genes had been tested under different experimental conditions (data obtained from OGEE v2). In each panel, the first bar represents the overall SGD when all genes were considered, while other bars represent recalculated SGDs after essential genes were excluded; each bar represents a dataset, with the number below (such as 422, 423, 424, 425 of the left-top panel) being the corresponding unique dataset ID (UID) in OGEE v2.

**Supplementary Figure 2**. The division of the genome into leading and lagging strands. Replication termination sites were calculated as the origin plus half the genome size.

**Supplementary Figure 3**. predicted SGDs (y-axis) in 1,552 bacterial genomes using overall skews and their correlation with the observed SGDs (x-axis). Each dot represents a genome, color-coded by genomic GC-content.

**Supplementary Figure 4.** GC contents of coding genes increase with expression abundance (proxied by tAI; see also Methods); however, when expression abundances are similar, genes on the leading and lagging strands have similar GC-contents. X-axis: expression abundance from low (1) to high (5).

# Supplementary Tables

**Supplementary Table 1**. Prokaryotic genomes and associated gene features used in this study.

**Supplementary Table 2**. The energetic costs of the 20 amino acids.
